# Early prediction of severe autoimmune encephalitis: development and validation of a model incorporating readily available lactate dehydrogenase

**DOI:** 10.3389/fimmu.2026.1800767

**Published:** 2026-07-09

**Authors:** Xin Ren, Lantao Liang, Yanbo Zhang, Lulu Zheng, Xiaoqing Li, Xiangmian Ma, Yanan Zhang, Ruohao Li, Kaixuan Bai, Xuejiao Qi, Yu Zhang, Xin Chen, Hui Bu

**Affiliations:** 1 Department of Neurology, The Second Hospital of Hebei Medical University, Shijiazhuang, Hebei, China; 2 Neurological Laboratory of Hebei Province, Shijiazhuang, Hebei, China; 3 Department of Neurology, People Hospital of Xingtai, Xintai, Hebei, China; 4 Department of Neurology, The Second Affiliated Hospital of XingTai Medical College, Xintai, Hebei, China; 5 Department of Neurology, Hebei Chest Hospital, Shijiazhuang, Hebei, China

**Keywords:** autoimmune encephalitis, biomarker, lactate dehydrogenase, nomogram, prediction model, severity

## Abstract

**Background:**

Autoimmune encephalitis (AE) is a severe neuroinflammatory disease with a substantial risk of progression to critical illness requiring intensive care. Early identification of patients at high risk of severe disease is essential but remains challenging because of heterogeneous presentations and the lack of objective, readily available prognostic tools. Lactate dehydrogenase (LDH), a ubiquitous enzyme associated with cellular injury and immune activation, has been linked to disease severity in systemic autoimmune disorders; however, its prognostic value in AE remains unexplored. This study aimed to develop a clinical prediction model for severe AE and to evaluate serum LDH as a core biomarker for prognosis and differential diagnosis.

**Methods:**

In this multicenter retrospective study, 299 adult patients with AE were analyzed. Severe AE was defined by intensive care unit admission or the presence of major disability (modified Rankin Scale ≥ 3). Independent predictors were identified using multivariable logistic regression and incorporated into a nomogram. The model underwent both internal and external validation. Serum LDH was additionally evaluated as a standalone biomarker by comparison with viral encephalitis (VE) controls (n = 243) and across AE antibody subtypes.

**Results:**

Elevated serum LDH, impaired consciousness at admission, and prodromal infection were identified as independent predictors of severe AE. The resulting nomogram demonstrated excellent discriminatory performance (area under the curve [AUC] 0.947 in the training cohort, 0.882 in the validation cohort) and good calibration. Serum LDH remained a robust predictor in the multivariable model. As a standalone predictor, LDH achieved an AUC of 0.887 for severe AE; an optimal cutoff value of 215 U/L yielded a sensitivity of 83.3% and a specificity of 84.1%. Notably, LDH levels were significantly higher in AE than in VE. Furthermore, elevated LDH demonstrated a significant positive correlation with the risk of severe disease across key AE subtypes, including anti-N-methyl-D-aspartate receptor, seronegative, anti-LGI1, anti-GAD65, and anti-GFAP encephalitis.

**Conclusion:**

This study presents a validated and readily applicable nomogram for early risk stratification in AE. Serum LDH emerges as a robust, accessible biomarker that supports both prognostic assessment and differential diagnosis, providing a simple objective threshold (215 U/L) to inform timely clinical decision-making.

## 1 Introduction

Autoimmune encephalitis (AE) is a rapidly progressive neuroinflammatory disorder mediated by pathogenic autoantibodies and is associated with severe neurological sequelae and mortality. The estimated annual incidence in adults is approximately 0.5–1 per 100,000 individuals (1). Clinical manifestations are heterogeneous, including seizures, psychiatric and behavioral abnormalities, impaired consciousness, autonomic dysfunction, and cognitive impairment. Although antibodies against neuronal or glial antigens such as N-methyl-D-aspartate receptor (NMDAR) and leucine-rich glioma-inactivated 1 (LGI1) have been well characterized, a substantial proportion of patients remain seronegative (2–4). Approximately 55% of patients require intensive care unit (ICU) admission, with reported mortality rates of 14.5–23% and up to 37% experiencing long-term functional impairment (2, 5–7). Secondary complications include infections related to immunosuppressive or antiepileptic therapy, multiorgan failure, and ventilator-associated pneumonia. These, along with delayed ICU admission, are linked to poor outcomes (5, 8). Therefore, early identification of patients at risk of severe progression is essential to facilitate timely treatment and appropriate allocation of critical care resources (7, 9, 10). However, the clinical assessment of AE severity still largely relies on neurological function scales, such as the Clinical Assessment Scale for Autoimmune Encephalitis, and subjective symptom observation, with a lack of objective, quantitative early prediction tools (11–13). Consistent with our findings, prior studies involving large AE cohorts have shown that clinical symptoms such as seizures and reduced levels of consciousness are significantly associated with disease severity (13). More recently, several biomarkers—including anemia, elevated cerebrospinal fluid (CSF) interleukin-17a levels, increased intrathecal immunoglobulin G synthesis, and peripheral inflammatory indices such as neutrophil-to-lymphocyte ratio and monocyte-to-lymphocyte ratio have been proposed as predictors of severe AE (14, 15). However, current biomarkers are limited by invasiveness, suboptimal diagnostic performance, or limited clinical accessibility, highlighting the need for a simple and reliable biomarker for early risk stratification. Evidence regarding serum LDH as an early predictor of severe autoimmune encephalitis (AE) remains limited, and no validated predictive models have incorporated LDH for early severity prediction.

Lactate dehydrogenase (LDH) is a key cytoplasmic enzyme involved in glycolysis and is released into the systemic circulation in response to tissue ischemia, hypoxia, injury, or extensive cellular necrosis. Elevated serum LDH has long been recognized as a marker of cellular damage and tissue metabolic stress and is associated with adverse outcomes in conditions such as acute decompensated heart failure, acute ischemic stroke, and respiratory failure (16–20). Beyond its role as a metabolic enzyme, LDH (particularly LDHA) has been implicated in immunometabolic reprogramming of immune cells through glycolytic pathways, including the “Warburg effect”. This pathway contributes to immune activation and tissue injury in autoimmune diseases (21–23). In rheumatoid arthritis, enhanced LDHA-mediated glycolysis supports pro-inflammatory immune responses and promotes tissue damage (24). Collectively, these findings suggest that LDH may be associated with inflammatory and tissue injury processes in autoimmune conditions; however, it is a non-specific biomarker and its role in autoimmune encephalitis remains unclear. The profound neuroinflammation, blood–brain barrier (BBB) disruption, and systemic metabolic stress characteristic of severe AE provide a strong pathophysiological rationale for elevated serum LDH levels in this condition. Nevertheless, despite extensive validation of LDH as a prognostic marker in oncology and systemic autoimmune disorders, its association with disease severity and early progression risk in AE—a condition characterized by localized inflammation of the central nervous system (CNS)—remains poorly defined, and its clinical significance warrants further exploration.

Against this background, the present study aimed to systematically identify independent risk factors for severe AE and to develop and validate a clinical prediction model incorporating serum LDH using a multicenter retrospective cohort. In addition, we sought to examine the differential expression of LDH across various AE antibody subtypes and to evaluate its potential utility in differentiating AE from viral encephalitis (VE). Overall, this study aimed to identify risk factors for severe AE, develop and validate a clinical prediction model incorporating serum LDH, and evaluate its potential role in differentiating AE from viral encephalitis.

## 2 Materials and methods

### 2.1 Study design

**Figure 1:**
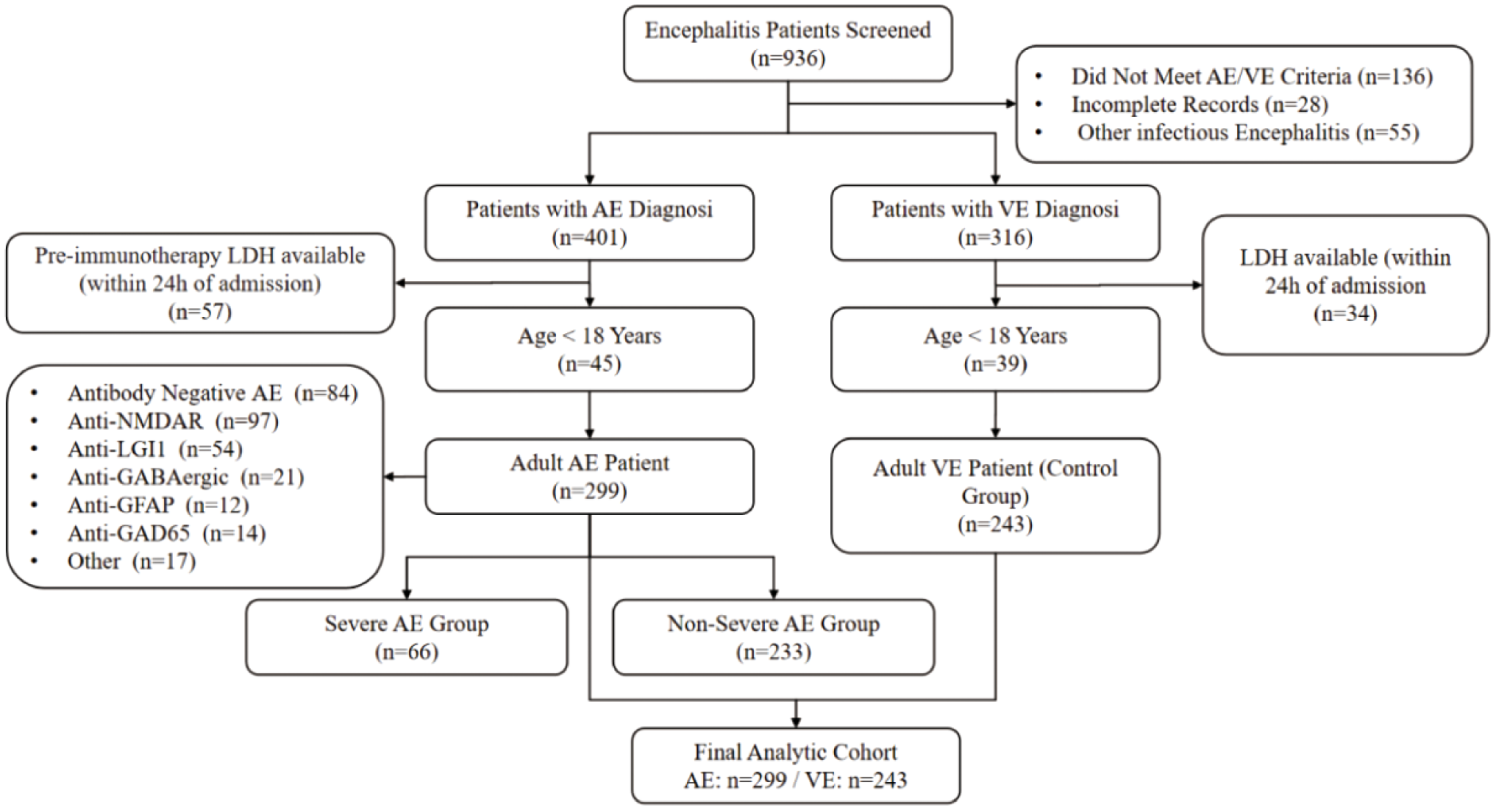
Flow diagram of patient screening, exclusion, and final cohort assignment in the autoimmune encephalitis prediction study. The diagram illustrates the consecutive identification of patients with encephalitis from three tertiary centers. After applying diagnostic and study eligibility criteria, 299 adult patients with autoimmune encephalitis (AE) and 243 adult patients with viral encephalitis were included in the final analytic cohort. Patients with AE were further classified as severe (n = 66) or non-severe (n = 233) based on intensive care unit admission or a modified Rankin Scale score ≥ 3. Key exclusion reasons are shown. The distribution of principal antibody subtypes within the AE cohort is indicated. VE, viral encephalitis; AE, autoimmune encephalitis; LDH, lactate dehydrogenase.

We consecutively enrolled adult patients diagnosed with AE who were admitted to the Neurology Departments of three tertiary hospitals in North China between August 2022 and August 2025. The diagnosis of AE was established in strict accordance with the clinical and laboratory criteria defined in the Chinese Expert Consensus on the Diagnosis and Treatment of Autoimmune Encephalitis. To evaluate the differential diagnostic value of the core biomarker, serum LDH, a comparator cohort of patients with VE was included. The diagnosis of VE was confirmed by CSF metagenomic next-generation sequencing, pathogen-specific antibody testing, or a combination of established clinical and CSF criteria. The patient screening process and cohort assembly are summarized in **Figure 1**.

This multicenter retrospective study was approved by the ethics committees of all participating centers, with waived informed consent for the anonymized retrospective data, and was performed in line with the Declaration of Helsinki.

### 2.2 Inclusion and exclusion criteria

Eligibility for the AE cohort required (1): fulfillment of established diagnostic criteria for AE (2); age ≥ 18 years (3); availability of a serum LDH measurement obtained within 24 h of admission and prior to initiation of immunotherapy; and (4) complete baseline clinical data. Patients were excluded if they had encephalitis of another confirmed etiology (e.g., bacterial, fungal, or parasitic) or concurrent severe systemic conditions known to substantially influence LDH levels, such as decompensated liver cirrhosis, hematologic malignancies, or active solid tumors. Similar exclusion principles were applied to the VE control cohort to ensure that LDH levels primarily reflected the infectious encephalitic process.

### 2.3 Definition of severe disease and cohort assignment

Patients with AE were classified according to disease severity based on the worst clinical status during hospitalization. The severe AE group was defined by the presence of either: (a) admission to the ICU for life-supportive treatment because of AE-related complications (including impairment of consciousness [Glasgow Coma Scale (GCS) ≤ 12], status epilepticus, status dystonicus, severe mental disorders, severe autonomic dysfunction causing hemodynamic instability, central hypoventilation syndrome, respiratory failure requiring ventilator support) or (b) an in-hospital modified Rankin Scale (mRS) score ≥ 3. The non-severe AE group comprised all other patients (mRS score < 3 and no ICU admission because of AE).

To ensure consistency across centers, all data collectors received standardized training on case criteria, each case was independently assessed by two raters, and disagreements were resolved by a third. An independent adjudication committee, blinded to biomarker results, centrally reviewed all clinical records and assessment scores.

### 2.4 Data collection

Clinical data were independently extracted from electronic medical records by two trained researchers. Collected variables included (1): demographic characteristics and clinical features at admission (2); serum LDH level (the primary predictor) and other routine inflammatory markers (e.g., C-reactive protein [CRP]) from the first blood sample obtained following admission (3); CSF parameters (4); findings from brain magnetic resonance imaging (MRI) and electroencephalography; and (5) anti-neuronal antibody profiles and initial immunotherapy regimens.

### 2.5 Statistical analysis

Statistical analyses were performed using R software (version 4.3.0). Continuous variables are presented as mean ± standard deviation or median (interquartile range), as appropriate, while categorical variables are expressed as frequencies and percentages. Missing data were handled using multiple imputation by chained equations (MICE) for variables included in model development (e.g., CRP and LDH), while variables not selected for prediction modeling were imputed using median values for descriptive analyses only; all imputations were performed prior to model fitting (**Supplementary Table 6**). Group comparisons were conducted using the Mann–Whitney U test, Kruskal–Wallis test with Dunn’s *post hoc* analysis and Bonferroni correction, chi-square test, or Fisher’s exact test, as appropriate. Variables with *P* < 0.10 in univariate analyses and with clinical or biological relevance were considered candidate predictors for multivariable logistic regression analysis. To reduce model complexity and minimize overfitting given the moderate sample size and limited number of severe events, variable selection was performed using a forward stepwise procedure based on the Akaike Information Criterion (AIC). The complete selection process and corresponding AIC values are provided in **Supplementary Table 4**. The final nomogram incorporated the selected core predictors together with clinically relevant variables (age and CRP) to enhance clinical applicability. Multicollinearity was assessed using variance inflation factors (VIFs), and all variables showed VIF values < 2, indicating low collinearity among predictors (**Supplementary Table 5**). The final model included five predictors, resulting in an events-per-variable ratio > 10, which is generally considered acceptable for logistic regression modeling. Model performance was evaluated in terms of discrimination, calibration, and clinical utility. Discrimination was assessed using the area under the receiver operating characteristic curve (AUC) and concordance index (C-index). Calibration was evaluated using calibration plots, calibration slope, and intercept. Clinical utility was assessed by decision curve analysis. Model validation was conducted in three stages (1): Internal validation was performed using bootstrap resampling with 1,000 iterations. In each bootstrap sample, the entire model development process, including variable selection and model fitting, was repeated. Model performance was evaluated in both bootstrap and original datasets, and optimism was estimated as the mean difference between these performances. The final optimism-corrected AUC was obtained by subtracting this optimism from the apparent AUC. This approach provides an unbiased estimate of model performance and reduces optimism associated with overfitting (2). random split-sample validation using a 70:30 training-test partition; and (3) independent external validation using data from a separate cohort. The predictive value of serum LDH as a standalone biomarker was evaluated using receiver operating characteristic (ROC) analysis to determine the optimal cutoff value based on the Youden index, along with corresponding sensitivity and specificity. Associations between LDH levels and severe disease risk across antibody subtypes were evaluated using subgroup-specific logistic regression models, and results were reported as odds ratios (ORs) with 95% confidence intervals. In addition, antibody subtype was not included in the primary candidate pool for the baseline prediction model to maintain a clinically parsimonious and widely applicable model. However, a sensitivity analysis including antibody subtype was conducted to evaluate its potential incremental value. Rare antibody subtypes with extremely small sample sizes were combined into an “other” category to ensure statistical interpretability. To account for multiple comparisons in subgroup analyses, *P*-values were adjusted using the Benjamini–Hochberg false discovery rate (FDR) procedure. In addition, an exploratory *post hoc* subgroup analysis stratified by seizure status was performed to assess whether the association between serum LDH levels and severe disease remained consistent in patients with and without seizures. This analysis was not part of the original prespecified statistical analysis plan and should therefore be interpreted as hypothesis-generating. The comparison of serum LDH levels between AE and VE was conducted as an exploratory, unadjusted analysis and was not adjusted for disease severity indicators. A two-sided *P* < 0.05 was considered statistically significant.

## 3 Results

### 3.1 Cohort derivation and patient characteristics

The patient screening and enrollment process is summarized in **Figure 1**. A total of 936 patients with suspected encephalitis were initially screened across the participating centers. After applying the diagnostic and study eligibility criteria, 401 patients were identified with AE, and 316 with VE. After excluding pediatric patients (age < 18 years), individuals without a serum LDH measurement obtained within 24 h of admission and prior to immunotherapy, and those meeting other predefined exclusion criteria, the final analytic cohort comprised 299 adult patients with AE and 243 adult patients with VE, who served as a disease control group for biomarker comparisons. Among the 299 patients with AE, 66 (22.1%) developed severe disease (defined as mRS ≥ 3) during hospitalization and were classified as the severe AE group, while 233 (77.9%) were classified as the non-severe group. The events-per-variable (EPV) ratio exceeded 10, supporting adequate sample size and reducing the risk of overfitting in logistic regression modeling. Baseline demographic and clinical characteristics of the two groups are presented in **Table 1**.

**Table 1:** Comparison of clinical characteristics between patients with non-severe and severe autoimmune encephalitis.

Variable	Non-severe(mRS<3) n = 233	Severe (mRS≥3) n = 66	*P* value
Age, years, median (IQR)	50.0 (32.0, 64.0)	44.0 (23.2, 59.8)	0.186
Male, n (%)	126 (54.1)	36 (54.5)	1.000
Comorbidities, n (%)			
Cancer History	13 (5.6)	6 (9.1)	0.455
Thyroid Dysfunction	67 (28.8)	26 (39.4)	0.134
Hypertension	54 (23.2)	20 (30.3)	0.306
Diabetes	35 (15.0)	9 (13.6)	0.933
Clinical features, n (%)			
HPI	66 (28.3)	39 (59.1)	**<0.001**
EBV Infection	49 (21.0)	15 (22.7)	0.899
MBA	92 (39.5)	30 (45.5)	0.466
Cognitive Impairment	103 (44.2)	23 (34.8)	0.223
Seizures	129 (55.4)	39 (59.1)	0.691
Consciousness Impairment	64 (27.5)	37 (56.1)	**<0.001**
Other neurological deficits	91 (39.1)	33 (50.0)	0.147
Sleep Disturbance	38 (16.3)	10 (15.2)	0.971
Fever	61 (26.2)	34 (51.5)	**<0.001**
Mixed Neuropsychiatric Type	82 (35.2)	28 (42.4)	0.352
Auxiliary examination, n/N (%)			
Brain MRI Abnormality	118/233 (50.6)	35/66 (53.0)	0.839
Positive Autoantibodies	53/111 (47.7)	17/36 (47.2)	1
Elevated Cytokines	17/29 (58.6)	13/15 (86.7)	0.121
Laboratory findings			
Serum Antibody Titer	131.19 ± 255.17 (111)	244.90 ± 351.09 (26)	0.322
CSF Antibody Titer	51.55 ± 124.13 (83)	81.62 ± 195.71 (25)	0.076
CRP, mg/L	8.53 ± 20.44 (202)	30.26 ± 40.96 (61)	**<0.001**
LDH, U/L	184.29 ± 45.98 (206)	313.08 ± 107.36 (62)	**<0.001**
CD3, %	74.29 ± 10.68 (51)	69.85 ± 12.46 (15)	0.215
CD4, %	1.89 ± 3.17 (48)	1.42 ± 0.40 (14)	0.686
CD16, %	12.24 ± 8.34 (48)	9.52 ± 6.81 (14)	0.266
CD19, %	12.74 ± 9.06 (50)	18.52 ± 13.15 (15)	0.168
CD37, %	31.40 ± 9.56 (48)	28.51 ± 6.22 (14)	0.229
CD38, %	39.80 ± 10.42 (49)	38.04 ± 10.19 (14)	0.677
CD45, cells/μL	1761.64 ± 816.25 (47)	1582.36 ± 936.48 (14)	0.229
IgG, g/L	15.55 ± 8.25 (124)	17.49 ± 9.78 (36)	0.427
IgA, g/L	2.31 ± 0.96 (124)	1.87 ± 0.84 (36)	**0.015**
IgM, g/L	0.96 ± 0.51 (124)	0.82 ± 0.45 (36)	0.099
C3, g/L	0.90 ± 0.18 (88)	0.90 ± 0.26 (28)	0.933
C4, g/L	0.23 ± 0.09 (88)	0.22 ± 0.10 (28)	0.907
CSF analysis			
White Blood Cell, cells/μL	39.31 ± 107.93 (187)	35.34 ± 68.44 (53)	0.187
Protein, g/L	0.62 ± 0.71 (189)	0.89 ± 1.00 (53)	**0.002**
Glucose, mmol/L	3.79 ± 1.40 (189)	3.86 ± 1.37 (52)	0.565
Chloride, mmol/L	123.49 ± 5.35 (189)	124.94 ± 6.18 (52)	**0.034**
Treatment, n (%)			
First-line Treatment	187 (80.3)	56 (84.8)	0.506
Second-line Treatment	18 (7.7)	15 (22.7)	**0.001**
Combination Therapy	18 (7.7)	14 (21.2)	**0.004**

Patients with autoimmune encephalitis (AE) were categorized into “non-severe” (modified Rankin Scale [mRS] < 3) and “severe” (mRS ≥ 3) subgroups based on admission to the intensive care unit for AE-related complications or in-hospital functional status. Data are presented as n (%) for categorical variables and mean ± standard deviation (number of patients with available data) for continuous variables, unless otherwise specified. *P*-values were calculated using the independent-samples *t* test for continuous variables and the χ² test or Fisher’s exact test for categorical variables, as appropriate. *P*-values in bold indicate statistical significance (*P* < 0.05).

AE, autoimmune encephalitis; mRS, modified Rankin Scale; HPI, history of prodromal infection; EBV, Epstein–Barr virus; MBA, mental and behavioral abnormalities; CRP, C-reactive protein; LDH, lactate dehydrogenase; CSF, cerebrospinal fluid; MRI, magnetic resonance imaging; Ig, immunoglobulin.

### 3.2 Univariate analysis: predictors of severe AE

As shown in **Table 1**, no significant differences were observed between the severe and non-severe AE groups with respect to age, sex, or the prevalence of major comorbidities, including malignancy, thyroid dysfunction, hypertension, and diabetes (all *P* > 0.05).

Clinical and auxiliary examination features: Clinically, a history of prodromal infection (HPI) (59.1% vs. 28.3%, *P* < 0.001), fever at disease onset (51.5% vs. 26.2%, *P* < 0.001), and impaired consciousness at admission (56.1% vs. 27.5%, *P* < 0.001) were significantly more frequent among the severe AE group. Other neurological manifestations, including mental or behavioral abnormalities, seizures, and cognitive impairment, did not differ significantly between groups. The prevalence of brain MRI abnormalities and autoantibody positivity was also comparable.

Laboratory biomarkers: Marked differences were observed in laboratory indicators of systemic inflammation and tissue injury. Serum LDH levels were substantially higher in patients with severe AE than in those with non-severe disease (313.08 ± 107.36 U/L vs. 184.29 ± 45.98 U/L, *P* < 0.001). Similarly, CRP concentrations were significantly elevated in the severe group (30.26 ± 40.96 mg/L vs. 8.53 ± 20.44 mg/L, *P* < 0.001). CSF analysis demonstrated higher protein levels in severe AE (0.89 ± 1.00 g/L vs. 0.62 ± 0.71 g/L, *P* = 0.002). In contrast, serum immunoglobulin A levels were markedly lower in patients with severe disease (1.87 ± 0.84 g/L vs. 2.31 ± 0.96 g/L, *P* = 0.015).

Treatment patterns: Consistent with greater disease severity, patients in the severe AE group required therapeutic escalation more frequently. Although the use of first-line immunotherapy was similar between groups, severe AE was associated with significantly higher rates of second-line immunotherapy (22.7% vs. 7.7%, *P* = 0.001) and combination intensive therapy (21.2% vs. 7.7%, *P* = 0.004).

### 3.3 Independent predictors of severe disease: multivariable analysis

To identify factors independently associated with the development of severe AE, multivariable logistic regression analyses were performed. The results are summarized in **Table 2**.

**Table 2:** Multivariable logistic regression analysis of factors associated with severe autoimmune encephalitis.

Model	Variable	Adjusted OR (95% CI)	*P* value
Full Model	Intercept	0.00 (0.00-0.00)	**<0.001**
	Age, per year	0.99 (0.97-1.01)	0.39
	Male (vs Female)	1.09 (0.49-2.41)	0.836
	CSF protein, per mg/L	0.94 (0.60-1.47)	0.782
	CRP, per mg/L	1.01 (0.99-1.02)	0.242
	LDH, per U/L	1.02 (1.02-1.03)	**<0.001**
	HPI	2.58 (0.94-7.08)	0.066
	Impaired consciousness	2.48 (1.15-5.33)	**0.02**
	Fever	0.92 (0.34-2.46)	0.862
Stepwise Model	Intercept	0.00 (0.00-0.00)	**<0.001**
	LDH, per U/L	1.02 (1.02-1.03)	**<0.001**
	HPI	2.71 (1.27-5.78)	**0.01**
	Impaired consciousness	2.52 (1.18-5.38)	**0.017**

The full model included all variables showing a univariate association with severe disease (modified Rankin Scale ≥ 3) at a significance level (*P* < 0.10). The stepwise model presents predictors retained after applying a forward stepwise selection procedure (retention criterion: *P* < 0.05). Odds ratios are reported per unit increase for continuous variables. The intercept represents the log odds of the outcome when all predictor variables are set to zero. For clinical interpretation, *P*-values in bold indicate statistical significance (*P* < 0.05). the OR for LDH corresponds to approximately 1.28 per 50 U/L increase and 1.73 per 100 U/L increase. OR, odds ratio; CI, confidence interval; mRS, modified Rankin Scale; CSF, cerebrospinal fluid; CRP, C-reactive protein; LDH, lactate dehydrogenase; HPI, history of prodromal infection. *P*-values in bold indicate statistical significance (*P* < 0.05).

Full model analysis: The initial full model included all candidate variables with a univariate association of *P* < 0.10, namely age, sex, CSF protein, CRP, LDH, HPI, impaired consciousness, and fever. After comprehensive adjustment, only two variables remained independently associated with severe AE: elevated serum LDH level (adjusted odds ratio [aOR] per 1 U/L increase = 1.02, 95% CI: 1.02–1.03; P < 0.001), corresponding to an OR of approximately 1.73 per 100 U/L increase, and impaired consciousness at admission (aOR = 2.53, 95% CI: 1.18–5.45; P = 0.017).

Parsimonious model derived by stepwise selection: To obtain a streamlined set of core predictors, a forward stepwise selection procedure was applied. The resulting parsimonious model retained three independent predictors: LDH (aOR per 1 U/L increase = 1.02, 95% CI: 1.02–1.03; P < 0.001, equivalent to an OR of approximately 1.73 per 100 U/L increase), HPI (aOR = 2.74, 95% CI: 1.29–5.84; P = 0.009), and impaired consciousness (aOR = 2.55, 95% CI: 1.20–5.44; P = 0.015).

Predictor selection for clinical nomogram construction: For development of a clinically applicable nomogram, we sought to balance statistical parsimony with clinical relevance. Accordingly, the final model incorporated the three core predictors identified by stepwise selection (LDH, HPI, and impaired consciousness). In addition, age and CRP were included in this final model based on their established clinical relevance to systemic disease severity, their significant associations in univariate analyses (**Table 1**), and the aim of enhancing the model’s clinical utility and acceptance, despite their lack of independent significance in the fully adjusted multivariable model.

### 3.4 Development and validation of a clinical prediction nomogram

**Figure 2:**
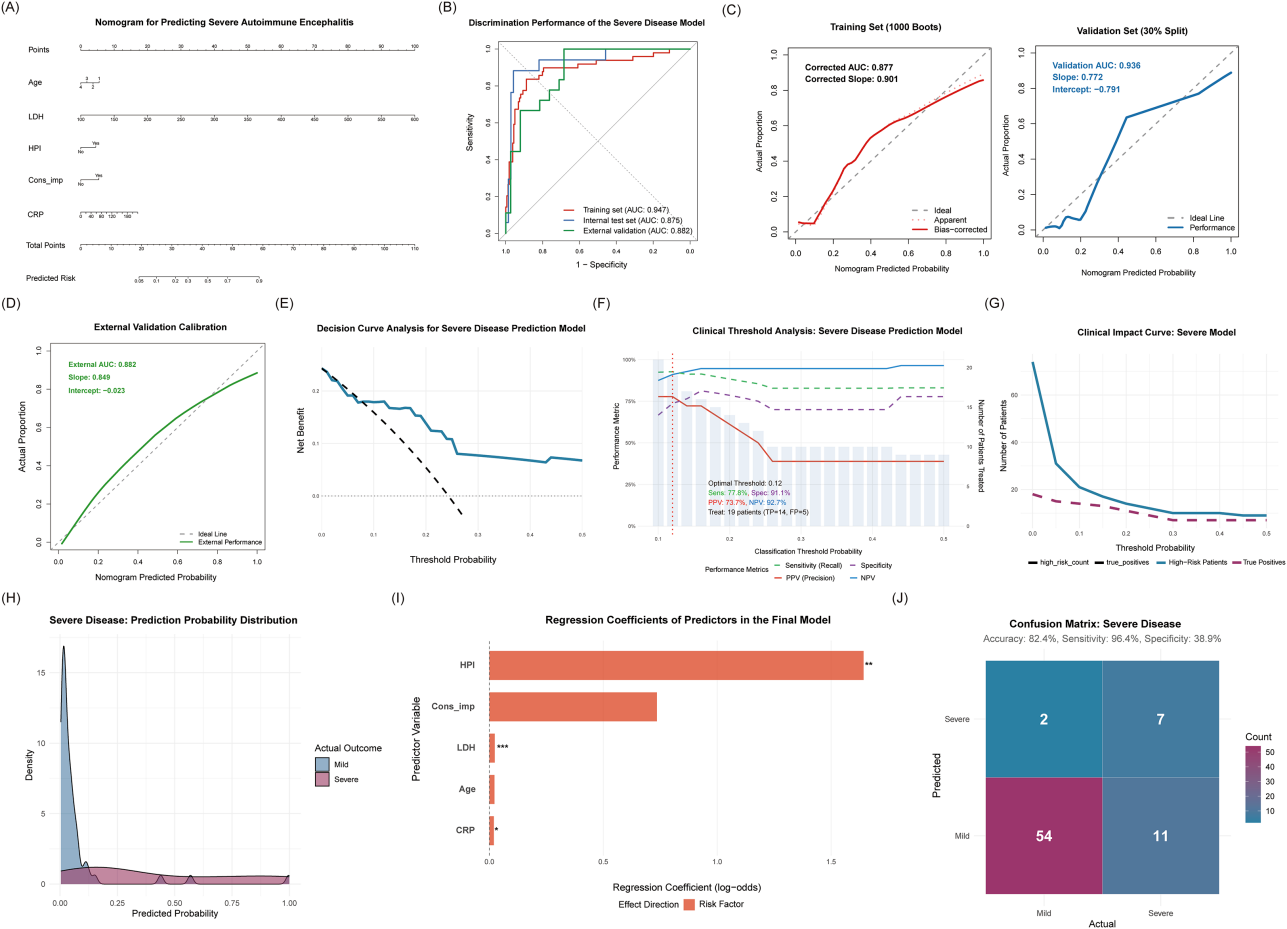
Development, validation, and clinical utility of the nomogram for predicting severe autoimmune encephalitis. **(A)** Clinical prediction nomogram: Points assigned to each predictor are summed to estimate an individual patient’s risk of severe autoimmune encephalitis (AE). **(B)** ROC curves: Discriminative performance in the training (AUC = 0.947), internal testing (AUC = 0.875), and external validation (AUC = 0.882) cohorts. **(C)** Internal calibration: ROC curves for 1000-fold bootstrap internal validation (AUC = 0.877) and 30% split-validation (AUC = 0.936). **(D)** External calibration: Agreement between predicted and observed outcomes in the external cohort (slope=0.849, intercept=-0.023). The dotted line represents ideal prediction. **(E)** Decision curve analysis showing that the nomogram (blue line) yields a higher net benefit than “treat-all” or “treat-none” strategies across a wide range of risk thresholds. **(F)** Performance metrics, including sensitivity, specificity, positive predictive value, and negative predictive value, across decision thresholds on the validation cohort. **(G)** Clinical impact curve illustrating concordance between the number of patients classified as high risk by the nomogram and the observed number of severe AE cases across thresholds. **(H)** Density distributions of predicted probabilities for patients with non-severe and severe outcomes. **(I)** Regression coefficients (log-odds) of predictors included in the final nomogram. **(J)** Confusion matrix of the nomogram’s predictive performance in the validation cohort at the optimal probability cutoff of 0.12. AE, autoimmune encephalitis; AUC, area under the curve; PPV, positive predictive value; NPV, negative predictive value; TN, true negative; FP, false positive; FN, false negative; TP, true positive; LDH, lactate dehydrogenase; HPI, history of prodromal infection; CRP, C-reactive protein.

A clinical prediction nomogram incorporating LDH, HPI, impaired consciousness, age, and CRP was developed to estimate the risk of severe AE (**Figure 2A**). The model demonstrated good discriminative performance across derivation and validation datasets. The apparent AUC was 0.947 in the training cohort and remained acceptable in the internal test cohort (AUC = 0.875) and external validation cohort (AUC = 0.882), indicating reasonable model generalizability despite the expected decline in performance from the derivation dataset (**Figure 2B**). To assess the incremental value of age and CRP, we compared the 5-variable nomogram with the 3-variable model (LDH, history of prodromal infection, and impaired consciousness). The 5-variable model showed only a minimal improvement in discrimination (AUC: 0.911 vs. 0.906), suggesting limited incremental value of age and CRP (**Supplementary Figure 1A**). Calibration analyses demonstrated good agreement between predicted and observed probabilities across validation datasets. Bootstrap internal validation using 1,000 resamples yielded an optimism-corrected AUC of 0.877 with a calibration slope of 0.901, indicating limited overfitting and good model stability. The optimism-corrected AUC showed only a minimal reduction compared with the apparent AUC. In the external validation cohort, the calibration curve remained close to the ideal reference line, with a slope of 0.849 and an intercept of −0.023 (**Figures 2C, D**). Decision curve analysis showed that the nomogram provided greater net clinical benefit than “treat-all” or “treat-none” strategies across a broad range of threshold probabilities (**Figure 2E**). At the optimal probability threshold of 0.12, the model achieved a sensitivity of 77.8%, specificity of 91.1%, positive predictive value (PPV) of 73.7%, and negative predictive value (NPV) of 92.7%, with an overall accuracy of 82.4% in the validation cohort (**Figures 2F, J**). Predicted probability distributions showed minimal overlap between severe and non-severe AE groups, further supporting the discriminatory capability of the model (**Figure 2H**). Although LDH had a smaller regression coefficient than HPI and impaired consciousness, it remained a significant predictor and was retained in the final model (**Figure 2I**). As a sensitivity analysis, LASSO penalized logistic regression with 10-fold cross-validation was performed. The λ.1se model retained LDH, history of prodromal infection, and impaired consciousness, which were fully consistent with the primary multivariable model, supporting the robustness of predictor selection (**Supplementary Figures 1B–D**).

### 3.5 Association of antibody subtypes with severe disease and subgroup analysis

**Figure 3:**
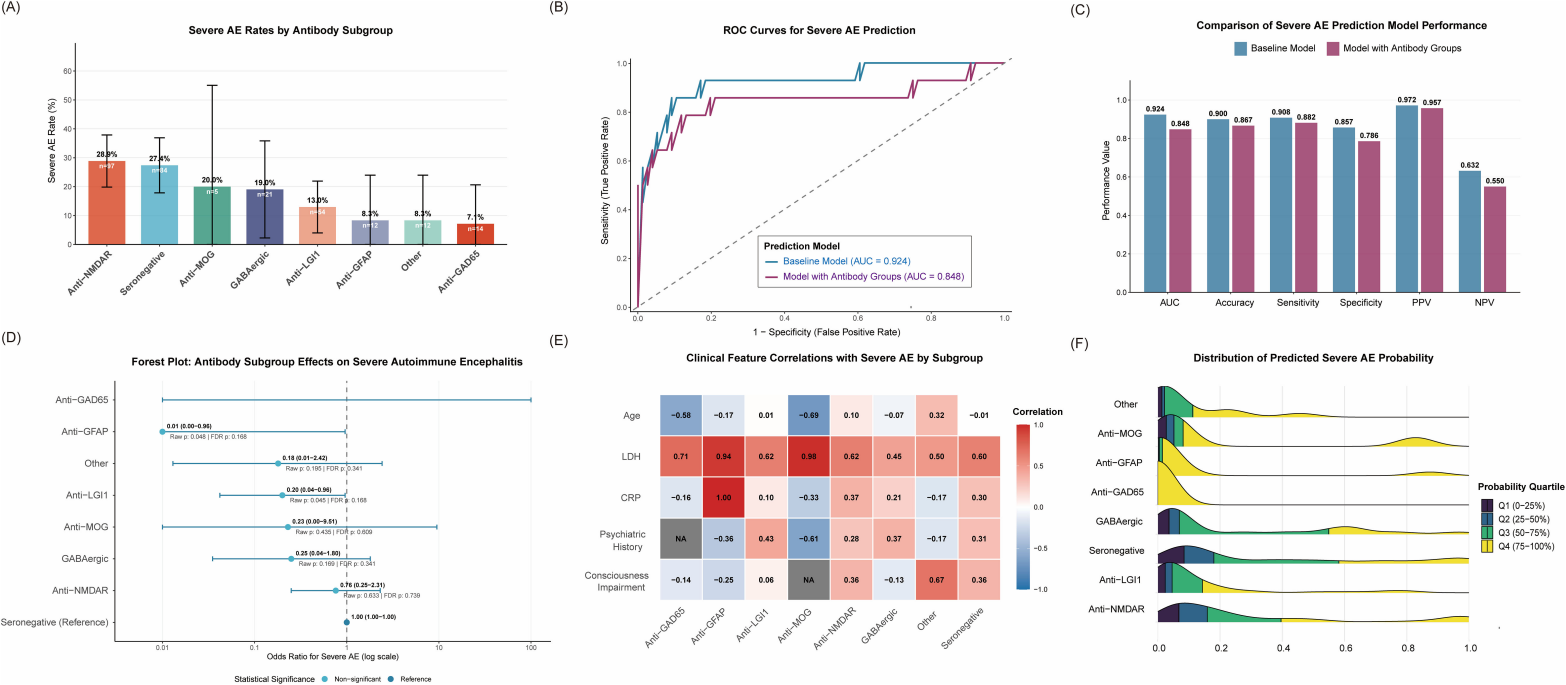
Association of antibody subtypes with severe autoimmune encephalitis and their impact on predictive model performance. **(A)** Incidence of severe disease across antibody subgroups, with error bars representing 95% confidence intervals. **(B)** Receiver operating characteristic curves comparing the baseline prediction model (area under the curve [AUC] = 0.924) with the antibody-subtype–enhanced model (AUC = 0.848). **(C)** Comparative performance metrics, including AUC, accuracy, sensitivity, specificity, positive predictive value, and negative predictive value, for both models in the test cohort (N = 90). **(D)** Forest plot showing adjusted odds ratios for severe disease across antibody subtypes, using seronegative autoimmune encephalitis as the reference. **(E)** Heatmap of Pearson correlation coefficients illustrating associations between clinical features and severe disease risk across antibody subtypes (red indicates positive correlations; blue indicates negative correlations). **(F)** Ridgeline plot showing distributions of predicted probabilities for severe disease across antibody subtypes, stratified by probability quartiles. AE, autoimmune encephalitis; ROC, receiver operating characteristic; AUC, area under the curve; OR, odds ratio; CI, confidence interval; PPV, positive predictive value; NPV, negative predictive value.

To examine heterogeneity in the risk of severe disease across antibody subtypes, we performed a predefined subgroup analysis. Baseline characteristics stratified by antibody subtype are summarized in **Supplementary Table 1**. Among antibody subtypes, anti-NMDAR and seronegative AE showed relatively higher proportions of severe disease (28.9% and 27.4%), whereas anti-LGI1, glial fibrillary acidic protein (GFAP)-associated, and other rare subtypes were less frequently severe (<10%) (**Figure 3A**). We further assessed whether incorporating antibody subtype information into the baseline prediction model—comprising LDH, HPI, and impaired consciousness—would improve predictive performance. Contrary to expectations, inclusion of antibody subtypes resulted in reduced discriminative ability, with the AUC dropping from 0.924 in the baseline model to 0.848 in the antibody-subtype–enhanced model (**Figure 3B**). Consistently, the baseline model outperformed the antibody-subtype–augmented model across all performance metrics (**Figure 3C**). In multivariable analysis with FDR correction, no antibody subtype remained independently associated with disease severity. Specifically, the initially observed associations for anti-GFAP astrocytopathy (aOR = 0.01, 95% CI: 0.00–0.96; adjusted *P* > 0.05) and anti-LGI1 encephalitis (aOR = 0.20, 95% CI: 0.04–0.96; adjusted *P* > 0.05) did not reach statistical significance following rigorous correction. Similarly, anti-NMDAR encephalitis showed no significant association (aOR = 0.76, 95% CI: 0.25–2.31; adjusted *P* = 0.739) (**Figure 3D**). Serum LDH showed a consistent positive association with disease severity across antibody subtypes, whereas CRP and impaired consciousness showed weaker and more heterogeneous associations (**Figure 3E**). In contrast, other variables, including CRP and impaired consciousness, showed weaker and more subtype-dependent associations with disease severity. Predicted risk distributions varied across antibody subtypes, with higher-risk profiles in anti-NMDAR and seronegative AE compared with GFAP and glutamic acid decarboxylase 65 (GAD65) subtypes (**Figure 3F**).

### 3.6 Serum LDH as a robust biomarker for disease severity

**Figure 4:**
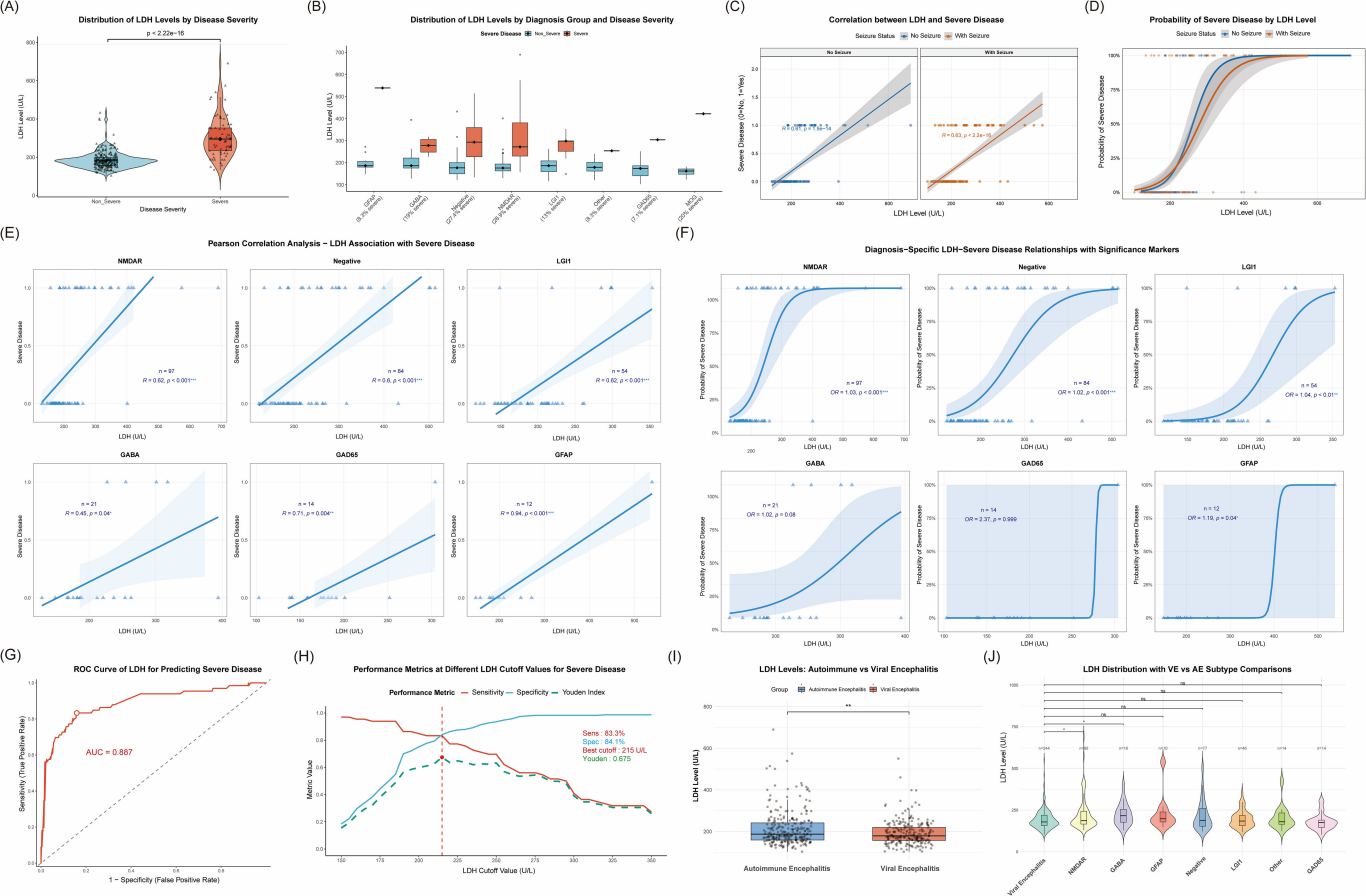
Comprehensive evaluation of serum lactate dehydrogenase as a biomarker of disease severity in autoimmune encephalitis. **(A)** Violin plot comparing serum lactate dehydrogenase (LDH) levels between patients with non-severe and severe autoimmune encephalitis (AE). **(B)** LDH levels across AE diagnostic subgroups, stratified by disease severity. **(C)** Correlation between LDH levels and risk of severe disease, stratified by seizure status (Pearson correlation coefficient [*r*] and *P*-value shown). **(D)** Calibration curves depicting the relationship between LDH levels and predicted probability of severe disease, stratified by seizure status. **(E)** Correlation between LDH levels and severe disease risk within major antibody subgroups, with sample size **(n)**, Pearson’s *r*, and *P*-value indicated for each subgroup. **(F)** Association curves with 95% confidence bands and adjusted odds ratios per 1 U/L increase in LDH for severe disease across antibody subgroups. **(G)** Receiver operating characteristic curve for LDH alone in predicting severe disease (area under the curve = 0.887). **(H)** Performance metrics, including sensitivity, specificity, and Youden index, across LDH cutoff values, with the optimal cutoff (215 U/L) highlighted. **(I)** Comparison of serum LDH levels between AE and viral encephalitis (VE) cohorts (**P < 0.01). **(J)** Comparison of LDH distributions between VE and AE antibody subtypes (*P < 0.05 vs. VE; ns, not significant). AE, autoimmune encephalitis; LDH, lactate dehydrogenase; mRS, modified Rankin Scale; ROC, receiver operating characteristic; AUC, area under the curve; OR, odds ratio; VE, viral encephalitis.

To further validate the central role of serum LDH in severe AE, we performed extensive subgroup and threshold analyses. LDH distributions differed markedly between non-severe and severe AE groups, with minimal overlap, as demonstrated in the violin plot (**Figure 4A**). This clear separation was consistent across all major diagnostic subtypes (e.g., anti-NMDAR, anti-γ-aminobutyric acid receptor [anti-GABABR]), with severe cases within each subtype uniformly exhibiting higher LDH levels than their non-severe counterparts (**Figure 4B**). LDH levels were positively associated with severe disease in both seizure and non-seizure groups (**Figure 4C**). Risk of severe disease increased with LDH levels, with a marked rise above approximately 200 U/L and high probabilities near 400 U/L (**Figure 4D**). Across antibody subtypes, LDH showed positive associations with disease severity, although estimates in rare subtypes should be interpreted with caution (**Figures 4E, F**). When evaluated as a standalone biomarker, LDH demonstrated excellent discriminatory performance for severe disease, with an AUC of 0.887 (**Figure 4G**). The Youden index identified an optimal LDH cutoff of 215 U/L, yielding a sensitivity of 83.3% and a specificity of 84.1% (**Figure 4H**). Finally, median LDH levels were higher in patients with AE than in those with VE in an exploratory unadjusted analysis (**Figure 4I**). This difference was primarily driven by patients with anti-NMDAR and anti-GABAB receptor antibody subtypes, whose LDH levels were significantly elevated relative to the VE cohort (**Figure 4J**).

## 4 Discussion

AE is increasingly recognized as a cause of severe neurological disability, with up to 87% of patients progressing to an mRS of 5 and frequently requiring early ICU admission for life-threatening complications (13, 25–28). Although multiple studies have explored predictors of severe AE—encompassing clinical manifestations, ancillary examinations, laboratory parameters, and scale-based assessments—the association between serum LDH levels at admission and disease severity remains unexplored (13). Clinically practical models for early risk stratification of severe AE remain limited, hindering timely clinical decision-making. We identified serum LDH as an independent predictor of severe AE and developed a nomogram for early risk stratification.

Elevated LDH levels have been consistently observed across a range of CNS disorders, including cerebral infarction, intracerebral hemorrhage, CNS infection, Guillain–Barré syndrome, and prolonged seizures (29, 30). LDH plays a key role in glycolysis, and its dysregulation has been associated with neuroinflammation and glial activation (31). Experimental studies have further demonstrated that enhanced LDHA activity promotes macrophage infiltration into the CNS and indirectly facilitates differentiation of CD4^+^ T cells into the T helper 17 subset, thereby exacerbating neuroinflammatory conditions such as multiple sclerosis (32–35). In the present study, serum LDH levels were higher in patients with AE than in those with VE, with particularly marked elevations observed in subgroups harboring antibodies against neuronal surface antigens, such as NMDAR and GABABR. This finding differs from prior reports suggesting no significant difference—or higher CSF LDH levels—in patients with intracellular antibodies or seronegative AE (36). These results suggest a possible association between serum LDH and disease context; however, the comparison between AE and VE should be interpreted as exploratory and may be influenced by differences in disease severity. Serum LDH levels were also associated with disease severity, with higher levels observed in severe AE. Risk of severe disease increased with higher LDH levels, consistent with prior evidence supporting LDH as a marker of disease severity across multiple pathological conditions (37). Although seizures are known to increase serum LDH levels, multivariable analyses in our cohort confirmed that elevated LDH remained an independent predictor of severe AE after adjusting for seizure occurrence (38, 39).

The pathogenesis of AE remains incompletely understood. Current evidence supports a central role for antibody-mediated synaptic dysfunction, while emerging data implicate cytotoxic T-cell–mediated neuronal injury (40). The higher LDH levels observed in AE compared with VE suggest potential differences between the two conditions; however, the underlying mechanisms remain unclear. Several hypotheses may explain LDH elevation in severe AE. First, severe systemic inflammation can induce endothelial dysfunction and microcirculatory impairment, for which LDH is a well-established biomarker (41). Second, systemic hypoxia resulting from severe AE-related complications—such as central hypoventilation or autonomic failure—may upregulate tissue LDH expression and enhance lactate production. This local accumulation of lactate may cause acidification of the immune microenvironment, dysregulating immune cell function and amplifying inflammatory responses (42–44). For example, enhanced CD8^+^ T-cell stemness and neutrophil activation may exacerbate AE, a hypothesis supported by evidence that higher proportions of peripheral CD8^+^ T cells correlate with increased disease severity (45, 46). Crucially, while LDH is a ubiquitous enzyme and a non-specific marker of tissue injury, its elevation in our cohort was associated with clinical severity (e.g., mRS scores) rather than inflammatory markers such as CRP. In multivariable analysis, serum LDH remained independently associated with disease severity in AE, suggesting that it may reflect overall disease burden and systemic physiological stress. Beyond LDH, our multivariable analysis identified prodromal infection and impaired consciousness as independent predictors of severe AE. In our cohort, 59.1% of patients with severe AE reported an HPI, consistent with prior studies implicating infection as a trigger or amplifier of autoimmune processes (5, 11, 47, 48). Notably, approximately 27% of patients with herpes simplex virus encephalitis develop AE within 3 months of antiviral therapy, potentially owing to BBB disruption and immune cell infiltration (49–51). Infection-related immune activation has also been described in other autoimmune neurological disorders (52–54). Impaired consciousness, reported in 71.7–86.4% of severe AE cases, is a well-recognized marker of advanced CNS involvement (12, 55). It has been identified as an independent risk factor for central hypoventilation in anti-NMDAR encephalitis and is associated with increased need for respiratory support (5, 56, 57). Impaired consciousness is also a known predictor of long-term cognitive dysfunction following severe sepsis (5, 58). The coexistence of prodromal infection and impaired consciousness may identify patients at increased risk of severe disease progression.

We developed a clinical prediction nomogram incorporating LDH, which showed good performance in identifying patients at risk of severe AE. Although only a limited number of AE severity prediction models have been reported, and differences in study design and outcome definitions limit direct comparison, our model demonstrated good discriminative performance in both internal and external validation (11, 13). Previous models have primarily relied on clinical manifestations, neurological assessment scales, or imaging findings. In contrast, our model additionally incorporates serum LDH, a routinely available laboratory biomarker that may provide complementary information for early risk stratification. In addition, several antibody-specific subgroup analyses, particularly for rare subtypes such as anti-GFAP and anti-GAD65, were based on small sample sizes and may have been underpowered. Consequently, these subgroup findings should be considered exploratory and interpreted with caution until validated in larger independent cohorts. The nomogram uses routinely available admission data, including medical history, physical examination findings, and serum LDH, to estimate risk of severe AE. Serum LDH remained an independent predictor in the final model and contributed to AE severity stratification alongside HPI and impaired consciousness. However, several limitations should be acknowledged. First, the retrospective design may have introduced residual confounding and information bias despite standardized data collection and central adjudication procedures. Second, although the model demonstrated favorable discrimination and calibration across internal and external validation datasets, the sample size remained moderate, particularly for severe AE cases. In addition, the use of stepwise regression for variable selection may increase the risk of model instability and overfitting. To mitigate this risk, we restricted candidate predictors to clinically plausible variables, maintained an acceptable events-per-variable ratio, and performed bootstrap, split-sample, and external validation analyses. A modest decline in performance in the external validation cohort was observed. Third, the external validation cohort was derived from participating centers within the same regional network, which may limit broader generalizability. Future studies should focus on large-scale prospective multicenter cohorts with more diverse populations and consider penalized regression methods to improve model robustness and generalizability. In addition, mechanistic studies are warranted to clarify the potential immunometabolic pathways linking LDH elevation to the progression of severe AE.

## 5 Conclusion

This study suggests that serum LDH may serve as a readily accessible biomarker associated with severe progression in autoimmune encephalitis (AE). Serum LDH levels were positively correlated with disease severity. LDH also showed potential utility in distinguishing AE from viral encephalitis (VE). A nomogram incorporating LDH and other clinical predictors demonstrated good discriminative performance and calibration in both internal and external validation cohorts. This suggests its potential value for early risk stratification in patients with AE. Future prospective multicenter studies with larger cohorts are warranted to validate these findings and to further elucidate the biological mechanisms underlying LDH elevation in AE.

## Data Availability

The raw data supporting the conclusions of this article will be made available by the authors, without undue reservation.
