# Supplementary figures and images for: Early prediction of severe autoimmune encephalitis: development and validation of a model incorporating readily available lactate dehydrogenase
Source: Front Immunol. 2026 Jul 9;17:1800767. doi: 10.3389/fimmu.2026.1800767 (PMC13391867; doi:10.3389/fimmu.2026.1800767)

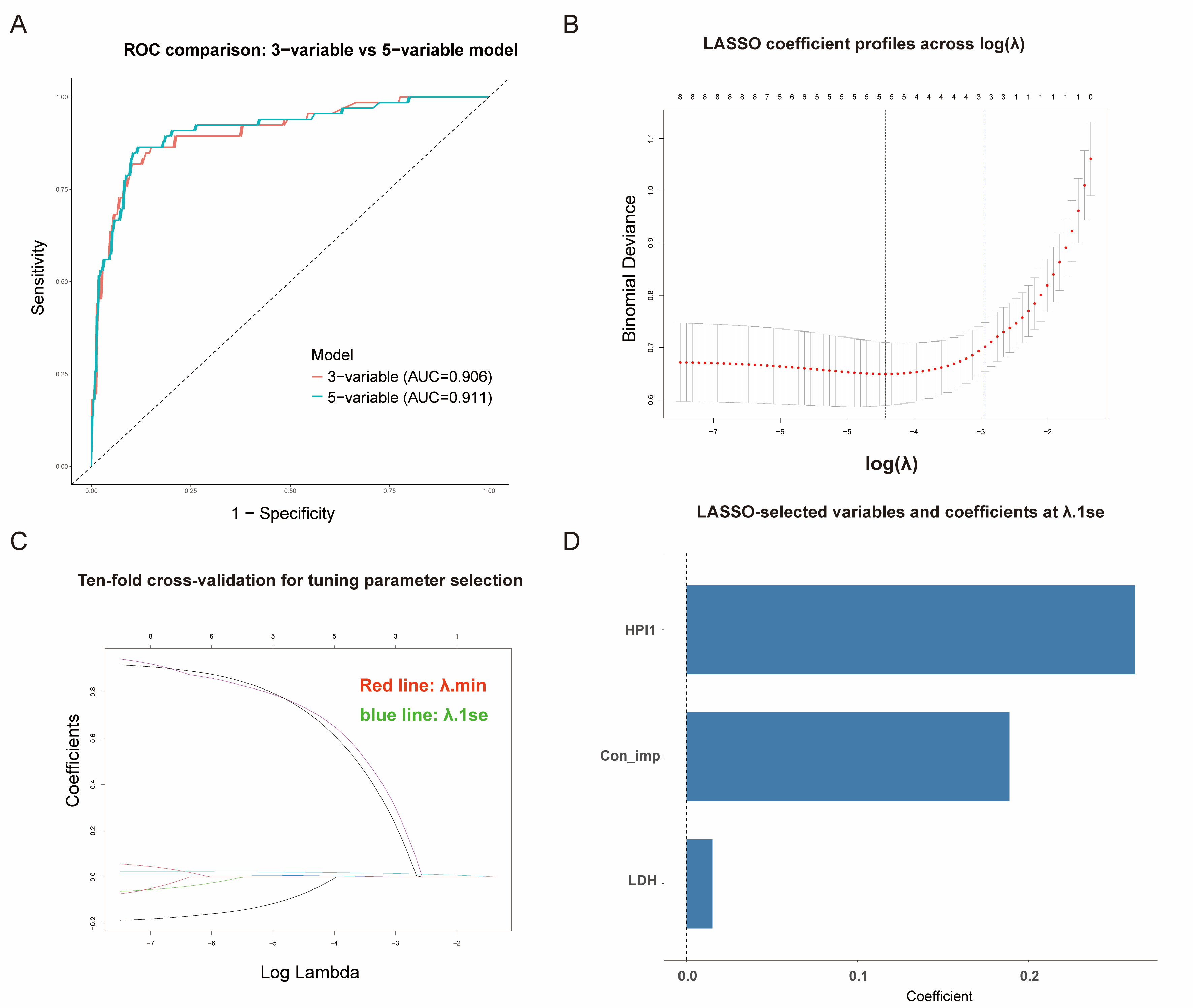

Supplement: Supplementary file 1 [file Image1.tif]
